# cDNA-AFLP analysis reveals differential gene expression in compatible interaction of wheat challenged with *Puccinia striiformis *f. sp. *tritici*

**DOI:** 10.1186/1471-2164-10-289

**Published:** 2009-06-30

**Authors:** Xiaojie Wang, Chunlei Tang, Gang Zhang, Yingchun Li, Chenfang Wang, Bo Liu, Zhipeng Qu, Jie Zhao, Qingmei Han, Lili Huang, Xianming Chen, Zhensheng Kang

**Affiliations:** 1 College of Plant Protection and Shaanxi Key Laboratory of Molecular Biology for Agriculture, Northwest A&F University, Yangling, Shaanxi, 712100, PR China; 2 USDA-ARS and Department of Plant Pathology, Washington State University, Pullman, WA 99164-6430, USA

## Abstract

**Background:**

*Puccinia striiformis *f. sp. *tritici *is a fungal pathogen causing stripe rust, one of the most important wheat diseases worldwide. The fungus is strictly biotrophic and thus, completely dependent on living host cells for its reproduction, which makes it difficult to study genes of the pathogen. In spite of its economic importance, little is known about the molecular basis of compatible interaction between the pathogen and wheat host. In this study, we identified wheat and *P. striiformis *genes associated with the infection process by conducting a large-scale transcriptomic analysis using cDNA-AFLP.

**Results:**

Of the total 54,912 transcript derived fragments (TDFs) obtained using cDNA-AFLP with 64 primer pairs, 2,306 (4.2%) displayed altered expression patterns after inoculation, of which 966 showed up-regulated and 1,340 down-regulated. 186 TDFs produced reliable sequences after sequencing of 208 TDFs selected, of which 74 (40%) had known functions through BLAST searching the GenBank database. Majority of the latter group had predicted gene products involved in energy (13%), signal transduction (5.4%), disease/defence (5.9%) and metabolism (5% of the sequenced TDFs). BLAST searching of the wheat stem rust fungus genome database identified 18 TDFs possibly from the stripe rust pathogen, of which 9 were validated of the pathogen origin using PCR-based assays followed by sequencing confirmation. Of the 186 reliable TDFs, 29 homologous to genes known to play a role in disease/defense, signal transduction or uncharacterized genes were further selected for validation of cDNA-AFLP expression patterns using qRT-PCR analyses. Results confirmed the altered expression patterns of 28 (96.5%) genes revealed by the cDNA-AFLP technique.

**Conclusion:**

The results show that cDNA-AFLP is a reliable technique for studying expression patterns of genes involved in the wheat-stripe rust interactions. Genes involved in compatible interactions between wheat and the stripe rust pathogen were identified and their expression patterns were determined. The present study should be helpful in elucidating the molecular basis of the infection process, and identifying genes that can be targeted for inhibiting the growth and reproduction of the pathogen. Moreover, this study can also be used to elucidate the defence responses of the genes that were of plant origin.

## Background

*Puccinia striiformis *Westend. f. sp. *tritici *Eriks., the causal fungus of stripe rust on wheat (*Triticum aestivum *L.), is a biotrophic obligate parasite. The pathogen is able to cause 100% yield loss [1] with frequent yield losses from 10 to 70% depending on cultivars grown and favorable weather conditions [2]. Leaf rust (*P. triticina *Eriks.) and stem rust (*P. graminis *Pers.:Pers. f. sp. *tritici *Eriks & E. Henn.) also cause similar damage to wheat production. In addition to their economic importance, rust fungi are particularly interesting because of their complex life cycle and the specialized infection structures. A great number of cytological studies have revealed details about spore attachment, germination, appressorium formation over host stomata and host invasion as reviewed by Hahn [3]. However, little is known about the mechanisms of biotrophic nutrient uptake [4,5]. The genetics, histology and pathology of host-rust pathogen interactions have been intensively explored over the last few decades [6]. The infection process of cereal rust fungi has been closely examined in several species, the molecular mechanisms of compatible and incompatible interactions between the host and pathogen, especially for the wheat-*P. striiformis *system, however, are poorly understood [7].

For successful parasitism of a fungal pathogen in plant living tissues, it is apparent that the pathogen has evolved strategies for manipulating host cellular metabolism, morphology and development [8,9,2]. Successful infection of a host plant by a pathogen requires the induction of a subset of pathogen genes that are essential for pathogenicity [10-12]. Less clear is the role and expression of specific host genes that may be required for successful pathogen infection of the plant. This is especially true for biotrophic rust fungi because they obtain nutrients and water completely from living plant tissues. Identification of both host and pathogen genes induced during a pathogen infection may provide insight into the compatible host-pathogen interaction at the molecular level.

Although a gene does not have to be up- or down-regulated to play a key role in a biological process, screening for differentially expressed genes is one of the most straightforward approaches to reveal the molecular basis of a biological system. As a differential screening method, cDNA-amplified fragment length polymorphism (cDNA-AFLP) is more stringent and reproducible than many other methods because it can amplify low-abundance transcripts [13]. The cDNA-AFLP technique is a robust, high-throughput, genome-wide expression tool for gene discovery [14-16], where prior knowledge of sequences is not required [17]. This technique has been further improved to avoid the possibility of several transcript derived fragments (TDFs) arising from a single gene/cDNA [18]. The technique should be especially useful for studying genes of the stripe rust pathogen because the fungus does not have known sexual reproduction and is difficult to culture on an artificial medium, which have limited the use of the traditional genetic and molecular techniques.

The objectives of this study were to apply the cDNA-AFLP technique to the wheat-stripe rust pathogen interaction pathosystem, identify genes regulated during the compatible interaction between the host and pathogen, and validate the expression patterns for some of the regulated genes through quantitative real-time polymerase chain reaction (qRT-PCR).

## Results

### Isolation of differentially expressed genes during interaction between wheat and the stripe rust fungus

**Figure 1:**
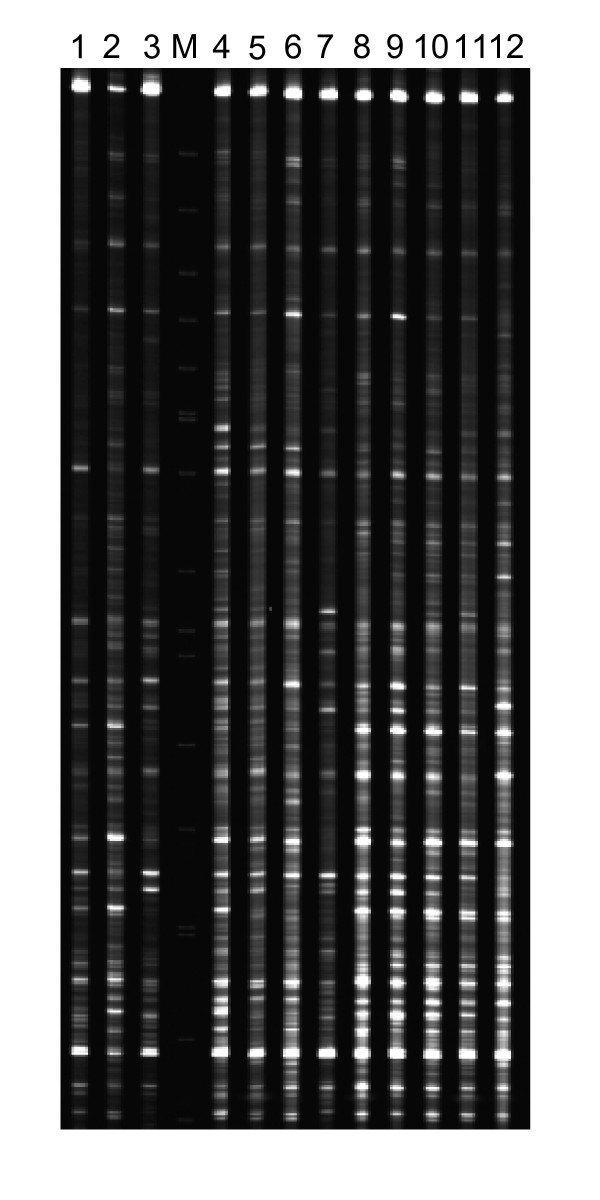
**Expression of wheat and *Puccinia striiformis *f. sp. *tritici *transcripts displayed by cDNA-AFLP**. An example showing selective amplification with primers MTG+TAG. Lanes 1 to 11: 6, 12, 18, 24, 36, 48, 72, 96, 120, 144 and 168 hpi, respectively; Lane 12: near 0 h (mock inoculation with sterile water); M: molecular weight marker.

The sampling time points ranging from 6 to 168 hpi corresponded to the different stages of stripe rust fungi infection processes, including spore germination (around 6 hpi), formation of substomal vesicle (8~12 hpi), infection hyphae (12~18 hpi), and haustorial mother cells and haustoria (18~24 hpi), proliferation of intercellular hyphae and numerous haustoria in host tissues (48~120 hpi), and generation of sporogenous cells at about 168 hpi, as described by Wang et al. [19]. High throughput coverage of leaf samples across time points during whole infection processes of stripe rust fungus yielded a large number of polymorphic bands using cDNA-AFLP analyses. The number of TDFs ranged from 58 to 85 per primer pair and their lengths were from 100 to 750 bp, depending upon primer combinations and time points. Figure 1 shows an example of the expression patterns. A total of 54,912 fragments were amplified with 64 primer combinations. The cDNA-AFLP fragments were highly reproducible as the band intensities were similar from the three biological replications for each time-point treatment. Altered expression patterns after inoculation were detected for 2,306 TDFs compared to the mock-inoculated plants near 0 hip, accounting for 4.2% of displayed fragments. Of the 2,306 TDFs, 966 were up-regulated and 1,340 down-regulated exclusively, indicating that more genes were suppressed during the compatible interaction. A total of 230 TDFs were selected for attempted further analyses, of which 215 were recovered from gels, re-amplified, sub-cloned and 208 of them were sequenced.

### Gene sequence analysis

**Figure 2:**
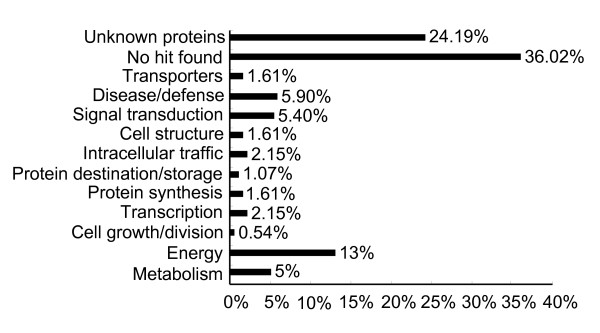
**Classification of differentially accumulated transcript derived fragments (TDFs) after inoculation with *Puccinia striiformis *f. sp. *tritici***. A total of 186 TDFs were classified based on the BLASTX homology search.

Of the 208 TDFs sequenced, 186 produced reliable (>100 bp) sequences. Each sequence was identified by similarity search using the basic local alignment search tool (BLAST) program against the GenBank non-redundant (nr) public sequence database. Sequences were classified into functional groups based on their homology with known proteins according to Bevan's method [20]. Sequence comparison of the 186 TDFs against the nr database revealed that the majority of them belonged to either no hit (36%) or unknown proteins (24%), and only 40% had putative functions. Among those TDFs with known functions, the majority were involved in the energy, signal transduction, disease/defense and metabolism groups (Figure 2). All of these unigenes were submitted to GenBank [see Additional file 1].

The results of BLAST searching database indicated that 32 (17%) of the sequenced 186 TDFs were likely from *P. striiformis*, and 87 (47%) were presumably from wheat, and the remaining 67 (36%) were unclear about their origin because they had no hits in public GenBank database. Of the 32 TDFs, 18 had significant homology (e < 1 × 10^-5^) at the amino acid level to *P. graminis *(Table 1). The encoding products of these genes included glycine dehydrogenase, fructose-1,6-bisphosphatase, UDP-glucuronic acid decarboxylase, ATP synthase subunit alpha, NADH-quinone oxidoreductase, glycosyl transferase, vesicular-fusion protein SEC17, plasma membrane proteolipid, glycine dehydrogenase and conserved hypothetical proteins.

**Table 1:** Transcript derived fragments (TDFs) from wheat leaves infected by *Puccinia striiformis *f. sp. *tritici *with homologies to genes in *P. graminis *f. sp. *tritici*

TDF	Accession No.	Size (bp)	*P graminis *f. sp. *tritici *clones	E-value
PST_72-1-2b	EF339818	235	PGTG_02587|Glycine dehydrogenase	7e-15
PST_C40	EF339683	311	PGTG_04973 | Fructose-1,6-bisphosphatase	1e-28
PST_C37^a^	EF339680	294	PGTG_01121| UDP-glucuronic acid decarboxylase	3e-22
PST_C38	EF339681	265	PGTG_15605| ATP synthase subunit alpha	3e-15
PST_C87	EF339730	237	PGTG_04870| ATP synthase subunit beta	1e-15
PST_C59^a^	EF339702	643	PGTG_06894 |NADH-quinone oxidoreductase chain 3	3e-74
PST_315-3^a^	EF339797	407	PGTG_16250| possible glycosyl transferase	1e-16
PST_84-3b^a^	EF339823	410	PGTG_13068|Conserved hypothetical protein	7e-25
PST_C81	EF339724	318	PGTG_08200 |Vesicular-fusion protein SEC17	3e-14
PST_C16	EF339660	366	PGTG_14848|Conserved hypothetical protein	2e-09
PST_C88	EF339731	216	PGTG_14274|Plasma membrane proteolipid 3	2e-12
PST_68b-1	EF339813	259	PGTG_18059|NADH-quinone oxidoreductase	4e-06
PST_68b-3^a^	EF339814	257	PGTG_18059|NADH-quinone oxidoreductase	7e-16
PST_C101^a^	EF339744	586	PGTG_07295|Conserved hypothetical protein	1e-21
PST_315-4^a^	EF339798	404	PGTG_10913|Predicted protein	1e-21
PST_C86^a^	EF339729	436	PGTG_15782|Hypothetical protein	8e-32
PST_C83^a^	EF339726	414	PGTG_02587 | Glycine dehydrogenase	7e-33
PST_C73	EF339716	249	PGTG_13068 | Conserved hypothetical protein	2e-24

^a ^These genes are confirmed to be of stripe rust fungus origin by sequencing.

### Identification of gene origins

**Figure 3:**
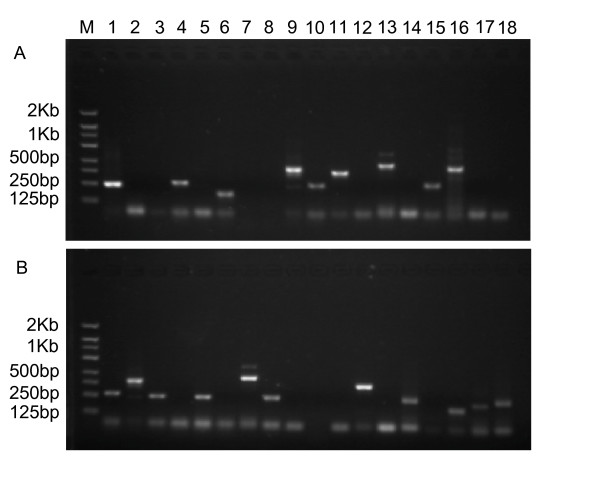
**PCR assays of genomic DNA from wheat (A) and *Puccinia striiformis *f. sp. *tritici *(B) using gene specific primers of the 18 TDFs that have high homologies with *P. graminis *genes**. DNA was extracted from wheat cv. 'Suwon 11' leaves and urediniospores of *P. striiformis *f. sp. *tritici *pathotype CY31. 20 ng DNA was subjected to PCR amplification using gene specific primers at annealing temperature of 53°C. The amplified products were analyzed using 1.5% agarose gel electrophoresis. Lane 1: PST_C40; 2: PST_315-3; 3: PST_84-3B; 4: PST_72-1-2B; 5: PST_C83; 6: PST_C16; 7: PST_315-4; 8: PST_C59; 9: PST_C38; 10: PST_68B-1; 11: PST_C81; 12: PST_C86; 13: PST_C87; 14: PST_C101; 15: PST_C73; 16: PST_C88; 17: PST_68B-3; 18: PST_C37; M: molecular weight markers.

To determine wheat or pathogen origin of the 18 TDFs with significant homology to genes in *P. graminis*, specific primers were designed for these genes to amplify genomic DNA from urediniospores of the stripe rust pathogen or wheat leaves. As showed in Figure 3, seven (PST-72-1-2b, PST-C16, PST-C38, PST-68B-1, PST-C81, PST-C87 and PST-C73) TDFs could be amplified from the genomic DNA of wheat leaves, while not from the pathogen, indicating that they were wheat genes. Other nine (PST-315-3, PST-84-3b, PST-83, PST-315-4, PST-C59, PST-86, PST-C101, PST-68B-3 and PST-C37) TDFs were successfully amplified from genomic DNA of the stripe rust pathogen, clearly showing that they were of *P. striiformis *origin. The remaining two (PST_c40 and PST_c88) could be amplified from both wheat and stripe rust pathogen. Sequencing analyses of the fragments amplified from both wheat and pathogen for PST_c40 and PST_c88 confirmed that the two TDFs were of wheat origin.

### Validation of expression patterns using qRT- PCR analyses

**Figure 4:**
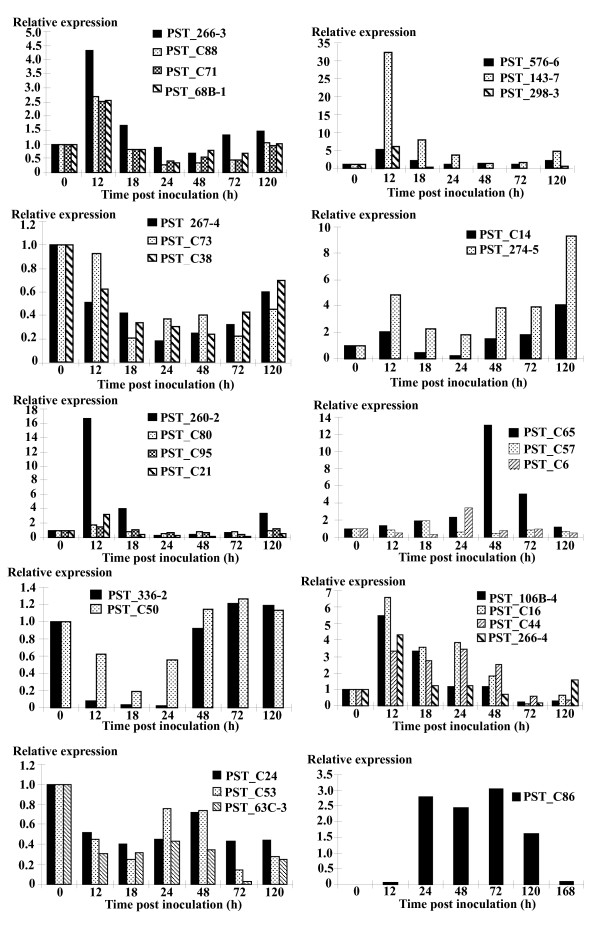
**Quantitative real-time PCR (qRT-PCR) analyses of 29 selected genes**. Leaf tissues were sampled for both inoculated and mock-inoculated plants at 12, 18, 24, 48, 72 and 120 hpi, as well as mock-inoculated plants near 0 hpi. Three independent biological replications were performed. The relative expression level for *Pst*-inoculated plants at each time point was calculated as fold of the mock-inoculated plants at that time point using the comparative ΔΔCT method. All data were normalized to the 18S rRNA expression level. The mean expression value was calculated for every transcript derived fragment (TDF) with three replications.

To investigate the reliability of cDNA-AFLP for detecting differentially expressed genes and verify the expression patterns observed in the cDNA-AFLP analyses, qRT-PCR analyses were carried out for 29 TDFs. These TDFs were selected based on their interesting cDNA-AFLP expression patterns in the time-course of the cDNA-AFLP experiment and homology to genes known to play a role in disease/defence, signal transduction or uncharacterized genes. The qRT-PCR analyses allowed us to monitor gene expression of both partners in the wheat-*P. striiformis *pathosystem at high levels of sensitivity and specificity. Expression profiles of the 29 TDFs in wheat leaves after inoculation are shown in Figure 4. For each TDF, the same expression pattern was found with qRT-PCR analyses as observed in the cDNA-AFLP tests, except for only one TDF (PST_336-2), presumably because of isolation of a wrong fragment.

As shown in Figure 4, TDFs involved in disease/defense (PST_266-3, PST_C88, PST_68B-1, PST_C71, PST_C44 and PST_266-4), signal transduction (PST_143-7, PST_576-6, PST_298-3, PST_C16 and PST_106B-4), and no hits (PST_260-2, PST_C80, PST_C95 and PST_C21) were strongly up-regulated as early as 12 hpi. Maximum induction of these genes occurred at 12 hpi and was followed by relatively constant expression. The transcripts of PST_260-2 and PST_143-7 increased by 16.68 and 32.49 fold, respectively. However, four genes (PST_63C-3, PST_C24, PST_C50 and PST_C53) related to energy were down-regulated as early as 12 hpi. PST_C57 and PST_C65 had maximum expression occurred at 18 and 48 hpi respectively. PST_C6 transcript showed maximum expression at 24 hpi. The accumulation of transcripts of PST_267-4, PST_C73, and PST_C38 decreased after 12 hpi. Both PST_C14 and PST_274-5 peaked as early as 12 hpi with maximum expression at 120 hpi. PST_C86 transcript encoding a fungal lipase was induced at 12 hpi. This gene peaked at 24 hpi and maintained high level of expression before 72 hpi, hereafter the expression decreased again and reached the same expression level as at 12 hpi by 168 hpi. Generally, majority of the 29 TDFs were induced and peaked at 12 to 24 hpi. These results suggested that the selected TDFs with putative functions in disease/defence or signal transduction might be triggered rapidly and play an active role during the early compatible interaction between wheat and the stripe rust pathogen.

In order to largely eliminate possible variations caused by experimental conditions, inoculation, plant development, or other factors, parallel mock inoculation with sterile water were included for each of all time points as control. We compared expression levels of all selected genes in the mock inoculated leaf samples across all time points and found that the 28 wheat genes did not change their expression level significantly without infection of the rust [see Additional file 2]. The results from the mock treatment at all time points support the conclusion that these genes changed their expression levels in response to the pathogen infection.

## Discussion

Lack of sexual reproduction and transformation system for the stripe rust pathogen has limited the use of most genetic and molecular techniques in studying genes of the pathogen involved in the interactions with its host plants. Interactions between the pathogen and its host plants have been studied almost restrictively by virulence analysis [1]. Genes of the pathogen had not been reported until recently. In 2007, Ling et al. [21] constructed a full-length cDNA library and reported the first group of 51 genes with putative functions for the stripe rust pathogen. Similarly, Zhang et al. [22] identified 267 genes with putative functions from a germinated urediniospore EST library. These studies did not address expression changes of the rust genes. Using the microarray technique, Coram et al. [23-25] identified genes involved in basal defense and different types of wheat resistance to stripe rust. However, the studies focused on plant genes using the wheat GeneChips and did not determine the pathogen genes involved in the compatible and incompatible interactions of the host and parasite. The present study was the first to determine expression of genes from both wheat and the stripe rust pathogen involved in a compatible interaction.

Using the cDNA-AFLP technique, we were able to detect 54,912 reproducible TDFs with 64 primer combinations. The number is within the same range as found in a previous study of the tomato-*Cladosporium fulvum *pathosystem, in which 50,000 TDFs were detected with all possible 1,024 primer combinations [15]. The fewer primer combinations producing more TDFs in the present study could be due to the larger genome size of wheat and the stripe rust fungus than those of tomato and *C. fulvum*. Nevertheless, the present study demonstrated that cDNA-AFLP is a powerful technique to study genes involved in the wheat-*P. striiformis *system. Because AFLP primers are universal, the cDNA-AFLP technique is applicable to any organisms or any host-pathogen systems for comprehensive transcript profiling [26]. In fact, the technique has been successfully employed to identify expressed genes in various plant-pathogen systems [15,16,27,28]. In comparison with microarray, cDNA-AFLP has low cost and does not require sequence information and expensive or sophisticated equipment. As showed in this study, cDNA-AFLP banding patterns are highly reproducible when compared with other techniques like differential display [29].

The time points used in the present study were selected based on our detailed microscopic study of the infection process (as previously described). Interestingly, 48% and 35% of the differentially expressed genes showed different degrees of change between 6–24 and 120–168 hpi, respectively. In contrast, only 17% of genes were differentially expressed at 48–96 hpi. The expression changes of these genes corresponded quite well to the different infection stages of the pathogen. Compared with the early stages of infection, the proportion of cDNA derived from the fungus increased slightly and that from the plant decreased at the late stages, as the fungal biomass increased. These results were similar to the expression patterns in compatible interaction between wheat and *P. triticina *[30] and were also supported by our histological study of compatible interaction between wheat and *P. striiformis *(data not shown). Functions could not be determined for many of the TDFs due to the fact that fungal genes were more limited in the database than plant genes. Therefore, the genes of no hit are more likely from the stripe rust fungus.

In the present study, we identified 89 genes which were up-regulated and 97 down-regulated in a compatible interaction. The up-regulated genes were remarkably similar to that of a recent study by Coram et al. [25]. They reported that 73 genes were up-regulated in a compatible interaction between wheat (cv. Avocet S) and a US wheat stripe rust pathotype (PST-78), which belonged to the major categories of functions as described in the present study. The expression of these genes also peaked at 24 hpi. In contrast, Coram et al. [25] only identified two genes down-regulated for the compatible interaction. Such difference might be due to the fact that the Affymetrix GeneChip does not include all possible wheat genes. The cDNA-AFLP technique does not have such limitation. Different wheat genotypes and stripe rust pathotypes also might contribute to the difference.

Ideally, the mock treatment should be used for all time-points in the cDNA-AFLP experiments. However, the increase of cost and time for such experiments could be substantial as we used a large number of time points to represent all key time points of stripe rust infection. To reduce the experimental cost, only the 0-hpi treatment was included in the cDNA-AFLP study. Thus, not all transcript changes of the 186 genes can be attributed to regulations by stripe rust infection. Other factors such as plant growth could affect expression of some of the genes. However, we obtained a 96.6% confirmation rate by testing 29 genes in experiments including the mock treatment for every time point using the qRT-PCR technique. With such a high percentage of genes showing consistent expression patterns in both cDNA-AFLP and qRT-PCR experiments, it is conceivable to conclude that the most of the genes identified using cDNA-AFLP are involved in the compatible interaction between the host and pathogen, although further studies are needed to determine their roles in the interaction.

In a plant-pathogen pathosystem, differentially expressed genes from either the plant or pathogen may interplay in the interaction. In this study, we determined the origins of 18 genes that had high homologies to genes of the stem rust pathogen. Interestingly, 50% of the tested genes were from the stripe rust pathogen and another 50% from the wheat host. The origin for two of the genes amplified in both wheat and the pathogen were determined from wheat by sequencing the PCR fragments. Similarly, some genes identified from the plant based on the BLAST results might be from the pathogen. This suggests that BLAST analysis should be used only as an indication of potential origins, but definite determination of gene origins from infected leaves should be from PCR amplifications of genomic DNAs from the pathogen and host separately, and sequencing comparisons become necessary if a gene is amplified from both organisms. Thus, further studies are needed to determine origins of most other genes identified in this study.

Because systems for transformation and expression of stripe rust genes have not been developed and the number of putative wheat genes was large, we used bioinformatical approach to determine putative functions of genes with altered expression patterns. Of 186 sequenced TDFs, 74 had relatively clear functions in various categories. These genes can help us in understanding molecular changes in the compatible interaction.

Clone PST_C38 was predicted to encode wheat ATPase. Its expression was generally down-regulated upon infection. ATPase was suggested to control stomatal apertures in guard cells [31,32]. The reduced transcript level of this gene in wheat leaves might indicate an attempt of the host to limit water loss by reducing stomatal openings. Clone PST_C71 was identified as a putative expansin and its transcripts accumulated rapidly during the rust infection. Expansin is a family of closely-related non-enzymatic proteins found in the plant cell wall with important roles in plant growth and response of plants to an adverse environment [33]. Several genes including ribulose bisphosphate carboxylase (PST_63C-3), phosphoribulokinase (PST_C24) and carbonic anhydrase (PST_C50) encoding enzymes in the Calvin cycle were down-regulated during the rust infection. Chlorophyll a-b binding proteins (PST_C53) associated with photosynthesis-related functions were strongly down-regulated during the early stage of the infection. Transcriptional down-regulation of photosynthesis-related genes has been reported for compatible interactions between soybean and *Phytophthora sojae *[34] and grapevine and powdery mildew [35]. Plants infected by biotrophic fungal pathogens, such as rusts and powdery mildews, reduce their photosynthetic rates, possibly as a result of increased invertase activity that causes carbohydrate accumulation [7].

A significant outcome of this study was the identification of ten TDFs with signal transduction functions, including serine/threonine kinase (PST_143-7), receptor kinase (PST_576-6, PST_298-3 and PST_274-5), phosphatase (PST_C16) and G-protein coupled receptor (PST_106B-4). The majority of these genes were up-regulated, indicating that they are essential in plant defense as previously reported [25,36]. Several genes encoding enzymes were up-regulated in infected leaves, including (1,3;1,4)-beta glucanase (PST_C44), peroxidase (PST_C6) and quinone reductase (PST_68B-1). This observation was similar to cases for salt-stress induced hydrophobic peptide (PST_C88), protease inhibitor (PST_266-3) and subtilisin-chymotrypsin inhibitor (PST_266-4) [37-39]. These genes have been assigned a function related to resistance in many pathosystems. This indicates the presence of a general, although weak, defense response in susceptible plants [25,40]. However, this was not the case for a disease resistance gene (PST_267-4), encoding nucleotide-binding site leucine-rich repeat (NBS-LRR) protein. The expression of this gene was down-regulated in the compatible interaction between wheat and the stripe rust fungus, which was in agreement with recently developed models [41].

Compared to TDFs of wheat origin as discussed above, relatively few genes were identified from the stripe rust pathogen, which could be due to higher abundance of wheat mRNA than fungal mRNA in the infected leaves. We clearly identified nine genes from *P. striiformis *f. sp. *tritici*. Because expressions of these genes were altered during the infection, they should be essential for the fungus to establish a compatible interaction with wheat plants. For example, ATPase was reported to reduce transpiration rates and stomatal apertures in rust fungus infections before uredia were fully developed [42]. The pathogen gene, TDF PST_C86, has homology to a lipase in *Aspergillus fumigatus*. Although little is known about the influence of lipases during infection of plants by fungi, intracellular lipases have been shown to be involved in appressorium formation of *Magnaporthe grisea *[43]. The first evidence of involvement of secreted lipases in plant infection came from Comménil et al. [44]. Eddine et al. [45] reported a secreted lipase in the pea pathogen *Nectria haematococca*. Voigt et al. [46] found that transformation-mediated disruption of *FGL1 *encoding a secreted *Fusarium graminearum *lipase led to reduced extracellular lipolytic activity in culture and reduced virulence to both wheat and maize. Because the lipase-like PST_C86 gene is from the stripe rust pathogen, its elevated expression during the infection process observed in this study suggests that the gene is important for the pathogen infection. More studies are needed to test the hypothesis and to determine the functions of the rust genes in the pathogen growth and interaction with wheat plants.

The present study was focused on a compatible interaction between the wheat and *P. striiformis *pathosystem to identify genes essential for establishing disease. A parallel study was undertaken to focus on incompatible interaction using the same wheat genotype and a pathotype of the pathogen avirulent to *YrSu *to identify genes essential for resistance and avirulence. As transformation and gene expression systems are currently under development in several laboratories, genes detected in the present study will be used in functional analysis for identifying genes involved in hypersensitive response, as done by Gabriël et al. [15] with the tomato-*C. fulvum *pathosystem.

## Conclusion

In this study, we successfully used the efficient cDNA-AFLP technique to determine gene expression patterns in a compatible interaction between wheat and the stripe rust pathogen. More than 2,300 TDFs with altered patterns of gene expressions during the plant-pathogen interaction were identified. A general correlation of gene expression with different stages of the infection process was observed. Sequence analysis of 186 TDFs identified genes involved in various molecular events during the compatible interaction. These genes and their putative functions provide insight in understanding the wheat-stripe rust compatible interaction, and provide candidate genes for future function analysis. This study identified the first group of the stripe rust pathogen genes involved in the infection process. Further research is needed to study the incompatible interaction and compare compatible and incompatible interactions to identify unique and common genes in the different interactions of the wheat-stripe rust pathosystem.

## Methods

### Biological materials and inoculation

Wheat genotype Suwon 11 and *P. striiformis *pathotype CY31 were the biological materials used for analyses of cDNA-AFLP and qRT-PCR analyses of the targeted genes. Suwon 11 was reported to contain the stripe rust resistance gene *YrSu *[47] and CY31 is highly virulent on Suwon 11. Plants were grown and maintained following the procedure described by Kang and Li [48]. Seven-day old seedlings were inoculated with fresh urediniospores of CY31 using a paintbrush. Parallel mock inoculation was also performed with sterile water. After inoculation, all plants, including the mock-inoculated plants near 0 hpi, were incubated for 24 h in a 100% humid chamber, and were subsequently transferred to a growth chamber at 15°C with a 16-hour photoperiod. Inoculated and mock inoculated leaf tissues were respectively sampled at 6, 12, 18, 24, 36, 48, 72, 96, 120, 144 and 168 h post-inoculation (hpi), as well as plants of the mock-inoculated plants near 0 hip, then immediately frozen in liquid nitrogen, and stored at -80°C prior to extraction of total RNA for cDNA-AFLP analyses. The time points were selected based on the microscopic study of the compatible interaction between Suwon 11 wheat and pathotype CY31 reported by Wang et al [19]. Plants were rated for symptom development 15 days after inoculation. Three independent biological replications were performed for each time point.

### RNA preparation, cDNA synthesis and cDNA-AFLP reaction

Total RNA was isolated from about 200 mg of the frozen wheat leaves using the Trizol™ extraction method (Invitrogen, Carlsbad, CA). RNA quality and integrity were determined by running 2 μL of total RNA in a formamide denaturing gel along with an RNA ladder (Invitrogen, Carlsbad, CA). And RNA quantity was tested using the NanoDrop™ 1000 spectrophotometer (Thermo Fisher Scientific, USA). Twenty micrograms of total RNA was used initially for the first strand synthesis, followed by the second strand synthesis using the SMART™ PCR cDNA Synthesis Kit (Clontech, BD) following the manufacturer's instruction.

About 100 ng of double-stranded cDNA was subjected to standard AFLP template production, cDNA-AFLP reaction was performed using the IRDye^® ^Fluorescent 800 AFLP expression analysis kit (LI-COR). The cDNA was first digested with *Taq*I for 2 h at 65°C, followed by digestion with *Mse*I for 2 h at 37°C. After completion, the mixture was incubated for 20 minutes at 80°C to inactivate the restriction enzymes, and then ligated to the *Taq*I and *Mse*I double stranded adapters at 20°C for 2 h. The pre-amplification reaction was carried out using 1/10 volume of the digested and ligated cDNA as template and primers corresponding to *Taq*I and *Mse*I adapters without selective bases using 20 cycles (94°C, 30 s for denaturation; 56°C, 1 min for annealing; and 72°C, 1 min for extension). Following the pre-amplification, the product was diluted 10 × with TE buffer (10 mM Tris-HCl and 1 mM EDTA, pH 8.0), and 2 μL was used for selective amplification with primers having selective nucleotides at the 3'-end (*Mse*I: AC, AG, CA, CT, GA, GT, TC or TG; IRDye 800-labeled *Taq*I: GA, GT, TC, TG, CT, CA, AG or AC) using a "touchdown" program (94°C, 30 s for denaturation; 65°C 30 s for annealing, 72°C 1 min for extension). For the following 12 cycles, the annealing temperature was reduced in each cycle by 0.7°C from the previous cycle. For next 23 cycles, 56°C was used for annealing. After all cycles were completed, the amplification products were kept in the cycler at 4°C until removal. A total of 64 primer pairs were tested. The AFLP products mixed with 5.0 μL of loading buffer to each reaction were heat-denatured for 3 min at 94°C. 1.0 μL of each denatured sample was loaded and run for 2 h in a 6.5% denaturing polyacrylamide sequencing gel (KB^Plus ^6.5% gel, LI-COR) with 0.5 × TBE electrophoresis buffer (0.089 M Tris-borate, 0.089 M boric acid, and 0.002 M EDTA) using the LI-COR DNA Analyzer (Set voltage to 1500 V, power to 40 W, current to 40 mA, and temperature to 45°C, Model 4300 DNA Analyzer, LI-COR).

### Isolation, re-amplification, cloning and sequencing of TDFs

Three microliters of each denatured sample was loaded and run for 1 h in the 8% denaturing polyacrylamide sequencing gel using the LI-COR DNA Analyzer, and then, the gel was scanned on the LI-COR Odyssey^® ^Infrared Imaging System (LI-COR). The polymorphic TDFs based on presence, absence or differential intensity were cut from the gel with a sharp razor blade, with maximum care to avoid any contaminating fragment(s), resolved in 30 μL 1 × TE buffer, performed three freeze-thaw cycles and centrifuged at 15,000 × *g *for 20 min at 4°C. Two microliters of the aliquot was used for re-amplification in a total volume of 25 μL using the same set of corresponding pre-amplification primers and the program as follows: 94°C 30 s, 56°C 1 min, 72°C 1 min, 20 cycles; 4°C hold. The PCR products were resolved in a 2% 1 × TAE-agarose gel, and each single band was isolated and eluted using the QIAEX1 II gel extraction kit (Qiagen Inc., Valencia, CA). All PCR amplifications were performed in a PTC-200 programmable thermal cycler (MJ Research Inc., Waltham, MA). *Taq *enzyme and buffers were from Promega Corp., unless otherwise mentioned.

The eluted TDFs were cloned into pGEM-Teasy vector (Promega Corp., Madison, WI) following the manufacturer's protocol. The single primer extension sequencing reaction was performed on the recombinant plasmids with the TDF inserts and/or on the purified TDFs using the BigDye Terminator cycle sequencing kit v3.1 (Applied Biosystems, Foster City, CA) with the T7 promoter primer. Sequencing products were purified with the EDTA/NaAC method (Bigdye Terminator V3.1 Cycle Sequencing Kit Protocol, Applied Biosystems). To verify the sequence for a band in the cDNA-AFLP, we sequenced at least three clones for each re-amplified TDF. Sequencing was carried out on an automated ABI Prism 3130-xl sequencer (Applied Biosystems, Foster City, CA) at the Shaanxi Key Laboratory of Molecular Biology for Agriculture, Northwest A&F University, Yangling, China.

### Sequence Analysis

In order to analyze large-scale EST data, we built one local stand-alone EST analysis platform. First, PHRAP package  was used to pre-process the EST data. ABI files derived from the ABI3130xl genetic analyzer were converted into txt files using PHRED. Then, vector sequences were eliminated by CROSS_MATCH. All ESTs without vector sequences, in the format of fasta, were clustered using CAP3  with 21 as the overlap parameter. The outputs were uniseqs, which contain contigs and singlets. Then we eliminated sequences of less than 100 bp using perl scripts from uniseqs. BLAST analysis was conducted to the filtered uniseqs with stand-alone BLAST package  using BLASTX as engine, NR  as database, and E-value of 1e-5 as the cut point for acceptance of similar functions. In addition, other individual fungal whole-genome sequence databases such as the stem rust database  were BLAST searched for identifying genes possibly from the stripe rust pathogen.

### Origin identification of genes homologous to genes in the stem rust pathogen

Genomic DNA was extracted from the urediniospores of CY31 and leaves of wheat cv. Suwon 11 according to the protocol described by Wang et al. [49]. Specific primers for genes possibly from the stripe rust pathogen were designed using the software of Primer Premier 5.0. Standard PCR amplification was performed separately with genomic DNA from the urediniospores and wheat leaves as templates. The PCR products were run on an agarose gel along with a molecular size marker (DL2000, TaKaRa Biotechnology Co., Ltd), followed by sequencing analyses of all amplified bands.

### qRT-PCR and data analyses

Leaf tissues were sampled for both *Pst*-inoculated and mock-inoculated plants at 12, 18, 24, 48, 72 and 120 hpi, as well as the mock-inoculated plants near 0 hpi. Transcript abundance was assessed with three independent biological replicates. Reverse transcription was performed on 1 μg RNA using the MMLV reverse transcriptase (Promega, Madison, WI) according to the manufacturer's instructions. Primers for real-time PCR were designed using the program Primer Express (Applied Biosystems, Forst City, CA) based on the interested cDNA sequence. Real-time PCR reaction mixtures contained 12.5 μL 2 × SYBR Green PCR MasterMix (Applied Biosystems), 10 pM of each primer, 2 μL template (10 × diluted cDNA from leaf samples) and sterile distilled water to a total volume of 25 μL. To standardize the data, the amount of target gene was normalized over the abundance of the constitutive wheat 18S rRNA (GenBank accession no. AY049040), or stripe rust pathogen actin gene (GenBank accession no. ES322227, for PST_C86 qRT-PCR). Thermal conditions were 50°C, 5 min; 95°C for 10 min followed by 40 cycles of 95°C for 15 s and 60°C for 1 min. To detect primer dimerisation or other artifacts of amplification, a melting-curve analysis was performed immediately after completion of the real-time PCR (95°C 15 s, 60°C 15 s, and then slowly increasing the temperature to 95°C at a 2% ramp rate, with continuous measurement of fluorescence). Reactions for *Pst*-inoculated and mock-inoculated plants at the above mentioned time points, as well as three non-template controls, were performed.

Quantification of gene expression was performed using a 7500 Real-Time PCR System (Applied Biosystems, Forst City, CA). Dissociation curves were generated for each reaction to ensure specific amplification. Threshold values (CT) generated from the ABI PRISM 7500 Software Tool (Applied Biosystems, Foster City, CA, USA) were employed to quantify relative gene expression using comparative 2^-ΔΔCT ^method [50]. Relative expression levels for each of the 29 selected genes in mock inoculated leaf tissues were also computed using the comparative CT method [50].

## Authors' contributions

XJW: designed experiments, analyzed data and wrote manuscript. CLT: conducted qRT-PCR and collected and analyzed data. GZ: analyzed data and prepared manuscript. YCL and CFW: provided assistance in various experiments. BL and ZPQ: conducted bioinformatical analysis. JZ and QMH: prepared samples and collected data. LLH: coordinated the experiments and data analyses. XMC: provided advices for experiments and revised manuscript. ZSK: conceived the project, designed the experiments and wrote manuscript. All authors read and approved the final manuscript.

## Supplementary Material

Additional file 1 **Transcript derived fragments**. Transcript derived fragments (TDFs) from *Puccinia striiformis *f. sp. *tritici *infected wheat leaves with altered expression patterns and their closest matches in the GenBank database. Click here for file

Additional file 2 **RT-PCR quantification of transcripts**. RT-PCR quantification of transcripts for the 28 seletced TDFs and 18s RNA in mock and non-inoculated leaf tissues of Suwon 11' wheat at various time-points after inoculation with pathotype CY31 of *Puccinia striiformis *f. sp. *tritici*. Click here for file

## References

[B1] Chen XM (2005). Epidemiology and control of stripe rust [*Puccinia striiformis *f. sp. *tritici*] on wheat. Can J Plant Pathol.

[B2] Saari EE, Prescott JM, Roelfs AP, Bushnell WR (1985). World distribution in relation to economic losses. The cereal rusts, Diseases, distribution, epidemiology, and control.

[B3] Hahn M, Kronstadt J (2000). The rust fungi: cytology, physiology and molecular biology of infection. Fungal Pathology.

[B4] Matthias H, Ulrike N, Christine S, Michael G, Kurt M (1997). A putative amino acid transporter is specifically expressed in haustoria of the rust fungus uromyces fabae. Mol Plant-Microbe Interact.

[B5] Struck C, Hahn M, Mendgen K (1996). Plasma membrane H+-ATPase activity in spores, germ tubes and haustoria of the rust fungus *Uromyces viciae*-*fabae*. Fungal Genet Biol.

[B6] Kolmer JA (1996). Genetics of resistance to wheat leaf rust. Annu Rev Phytopathol.

[B7] Scholes JD, Lee PJ, Horton P, Lewis DH (1994). Understanding changes in the photosynthetic and carbohydrate metabolism of barley leaves with powdery mildew. New Phytol.

[B8] Chou HM, Bundock N, Rolfe SA, Scholes JD (2000). Infection of *Arabidopsis thaliana *with *Albugo candida *(white blister rust) causes a reprogramming of host metabolism. Mol Plant Pathol.

[B9] Clancy FG, Coffey MD (2000). Patterns of translocation, changes in invertase activity, and polyol formation in susceptible and resistant flax infected with the rust fungus *Melampsora lini*. Physiol Plant Pathol.

[B10] Hahn M, Mendgen K (1997). Characterization of in planta-induced rust genes isolated from a haustorium-specific cDNA Library. Mol Plant-Microbe Interact.

[B11] Hahn M, Neef U, Struck C, Göttfert M, Mendgen K (1997). A putative amino acid transporter is specifically expressed in haustoria of the rust fungus *Uromyces fabae*. Mol Plant-Microbe Interact.

[B12] Rogers LM, Flaishman MA, Kolattukudy PE (1994). Chitinase gene disruption in *Fusarium solani *f.sp. *pisi *decreases its virulence on pea. Plant Cell.

[B13] Lievens S, Gooriachtig S, Holsters M (2001). A critical evaluation of differential display as a tool to identify genes involved in legume nodulation: looking back and looking forward. Nucl Acids Res.

[B14] Breyne P, Zabeau M (2001). Genome-wide expression analysis of plant cell cycle modulated genes. Curr Opin Plant Biol.

[B15] Gabriëls SHEJ, Takken FLW, Vossen JH, de Jong CF, Liu Q, Turk SCHJ, Wachowski LK, Peters J, Witsenboer HMA, de Wit PJGM, Joosten MHAJ (2006). cDNA-AFLP combined with functional analysis reveals novel genes involved in the hypersensitive response. Mol Plant-Microbe Interact.

[B16] Polesani M, Desario F, Ferrarini A, Zamboni A, Pezzotti M, Kortekamp A, Polverari A (2008). cDNA-AFLP analysis of plant and pathogen genes expressed in grapevine infected with *Plasmopara viticola*. BMC Genomics.

[B17] Ditt RF, Nester EW, Comai L (2001). Plant gene expression response to *Agrobacterium tumefaciens *. Proc Natl Acad Sci USA.

[B18] Breyne P, Dreesen R, Cannoot B, Rombaut D, Vandepoele K, Rombauts S, Vanderhaeghen R, Inze' D, Zabeau M (2003). Quantitative cDNA-AFLP analysis for genome-wide expression studies. Mol Genet Genomics.

[B19] Wang CF, Huang LL, Buchenauer H, Han QM, Zhang HC, Kang ZS (2007). Histochemical studies on the accumulation of reactive oxygen species (O_2_^- ^and H_2_O_2_) in the incompatible and compatible interaction of wheat–*Puccinia striiformis *f.sp. *tritici*. Physiol and Mol Plant Pathol.

[B20] Bevan M, Bancroft I, Bent E, Love K, Goodman H, Dean C, Bergkamp R, Dirkse W, Staveren MV, Stiekema W, Drost L, Ridley P, Hudson SA, Patel K, Murphy G, Poffanelli P, Wedler H, Wedler E, Wambutt R, Weitzenegger T, Pohl TM, Terryn N, Geilen J, Villarroel R, Clerck RD, Montagu MV, Lecharny A, Auborg S, Gy I, Kreis M, Lao N, Kavanagh T, Hempei S, Kotter P, Entian KD, Rieger M, Schaeffer M, Funk B, Mueller-Auer S, Silvey M, James R, Montfort A, Pons A, Puigdomenech P, Douka A, Voukelatou E, Milioni D, Hatzopoulos P, Piravandi E, Obermaier B, Hilber H, Düsterhöft A, Moores T, Jones JD, Eneva T, Palme K, Benes V, Rechman S, Ansorge W, Cooke R, Berger C, Delseny M, Voet M, Volckaert G, Mewes HW, Klosterman S, Schueller C, Chalwatzis N (1998). Analysis of 1.9 Mb of contiguous sequence from chromosome 4 of *Arabidopsis thaliana*. Nature.

[B21] Ling P, Wang MN, Chen XM, Campbell KG (2007). Construction and characterization of a full-length cDNA library for the wheat stripe rust pathogen (*Puccinia striiformis *f.sp. *tritici*). BMC Genomics.

[B22] Zhang YH, Qu ZP, Zheng WM, Liu B, Wang XJ, Xue XD, Xu LS, Huang LL, Han QM, Zhao J, Kang ZS (2008). Stage-specific gene expression during urediniospore germination in *Puccinia striiformis *f. sp. *tritici*. BMC Genomics.

[B23] Coram TE, Settles ML, Chen XM (2008). Transcriptome analysis of high-temperature adult-plant resistance conditioned by *Yr39 *during the wheat-*Puccinia striiformis *f. sp. *tritici *interaction. Mol Plant Pathol.

[B24] Coram TE, Settles ML, Wang MN, Chen XM (2008). Surveying expression level polymorphism and single-feature polymorphism in near-isogenic wheat lines differing for the *Yr5 *stripe rust resistance locus. Theor Appl Genet.

[B25] Coram TE, Wang MN, Chen XM (2008). Transcriptome analysis of the wheat-*Puccinia striiformis *f.sp. *tritici *interaction. Mol Plant Pathol.

[B26] Bachem CWB, Hoeven RS van der, de Bruijn SM, Vreugdenhil D, Zabeau M, Visser RGF (1996). Visualisation of differential gene expression using a novel method of RNA fingerprinting based on AFLP: Analysis of gene expression during potato tuber development. Plant J.

[B27] Eckey C, Korell M, Leib K, Biedenkopf D, Jansen C, Langen G, Kogel KH (2004). Identification of powdery mildew-induced barley genes by cDNA-AFLP: Functional assessment of an early expressed MAP kinase. Plant Mol Biol.

[B28] Qin Ling, Overmars Hein, Helder Johannes, Popeijus Herman, Voort Jeroen Rouppe van der, Groenink Wouter, van Koert Paul, Schots Arjen, Bakker Jaap, Smant Geert (2000). An Efficient cDNA-AFLP-Based Strategy for the Identification of Putative Pathogenicity Factors from the Potato Cyst Nematode *Globodera rostochiensis*. MPMI.

[B29] McClelland M, Mathieu-Daude F, Welsh J (1995). RNA fingerprinting and differential display using arbitrarily primed PCR. Trends in Genetics.

[B30] Zhang L, Meakin H, Dickinson M (2003). Isolation of genes expressed during compatible interactions between leaf rust (*Puccinia triticina*) and wheat using cDNA-AFLP. Mol Plant Pathol.

[B31] Assmann SM, Simoncini L, Schroeder JI (1985). Blue light activates electrogenic ion pumping in guard cell protoplasts of *Viciafaba*. Nature.

[B32] Sze H, Li X, Palmgren MG (1999). Energization of plant cell membranes by H+-pumping ATPases. Regulation and biosynthesis. Plant Cell.

[B33] Cosgrove DJ (2000). Loosening of plant cell walls by expansins. Nature.

[B34] Moy P, Qutob D, Chapman BP, Atkinson I, Gijzen M (2004). Patterns of gene expression upon infection of soybean plants by *Phytophthora sojae*. Mol Plant-Microbe Interact.

[B35] Fung RW, Gonzalo M, Fekete C, Kovacs LG, He Y, Marsh E, McIntyre LM, Schachtman DP, Qiu W (2008). Powdery mildew induces defense oriented reprogramming of the transcriptome in a susceptible but not in a resistant grapevine. Plant Physiol.

[B36] Garcia-Brugger A, Lamotte O, Vandelle E, Bourque S, Lecourieux D, Poinssot B, Wendehenne D, Pugin A (2006). Early signaling events induced by elicitors of plant defenses. Mol Plant-Microbe Interact.

[B37] Dangl JL, Jones JGD (2001). Plant pathogens and integrated defense responses to infection insight review articles. Nature.

[B38] Dunaevsky YE, Elpidina EN, Vinokurov KS, Belozersky MA (2005). Protease inhibitors in improvement of plant resistance to pathogens and insects. Mol Biol.

[B39] Mukai H, Munekata E, Higashijima T (1992). G protein antagonists. A novel hydrophobic peptide competes with receptor for G protein binding. J Biol Chem.

[B40] Lin H, Doddapaneni H, Takahashi Y, Walker MA (2007). Comparative analysis of ESTs involved in grape responses to *Xylella fastidiosa *infection. BMC Plant Biol.

[B41] Belkhadir Y, Subramaniam R, Dangl JL (2004). Plant disease resistance protein signaling: NBS-LRR proteins and their partners. Curr Opin Plant Biol.

[B42] Tissera P, Ayres PG (1986). Transpiration and the water relations of faba bean (*Vicia faba*) infected by rust (*Uromyces viciae-fabae*). New Phytol.

[B43] Thines E, Weber RW, Talbot NJ (2000). MAP kinase and protein kinase a-dependent mobilization of triacylglycerol and glycogen during appressorium turgor generation by *Magnaporthe grisea*. Plant Cell.

[B44] Comménil P, Belingheri L, Bauw G, Dehorter B (1999). Molecular characterization of a lipase induced in *Botrytis cinerea *by components of grape berry cuticle. Physiol Mol Plant Pathol.

[B45] Eddine AN, Hannemann F, Schäfer W (2001). Cloning and expression analysis of *NhL1*, a gene encoding an extracellular lipase from the fungal pea pathogen *Nectria haematococca MPVI *(*Fusarium solani *f. sp. *pisi*) that is expressed in planta. Mol Genet Genomics.

[B46] Voigt CA, Schäfer W, Salomon S (2005). A secreted lipase of *Fusarium graminearum *is a virulence factor required for infection of cereals. The Plant Journal.

[B47] Cao ZJ, Jing JX, Wang MN, Shang HS, Li ZQ (2003). Relation analysis of stripe rust resistance gene in wheat important cultivar Suwon 11, Suwon 92 and Hybrid 46. Acta Bot Boreal-Occident Sin.

[B48] Kang ZS, Li ZQ (1984). Discovery of a normal T. type new pathogenic strain to Lovrin10. Acta Cllegii Septentrionali Occidentali Agriculturae.

[B49] Xiaojie W, Wenming Z, Buchenauer H, Jie Z, Qingmei H, Lili H, Zhensheng K (2008). Development of a PCR-based detection of Puccinia striiformis in latent infected wheat leaves. European Journal of Plant Pathology.

[B50] Livak KJ, Schmittgen TD (2001). Analysis of relative gene expression data using real-time quantitative PCR and the 2^-ΔΔCT ^method. Methods.

