# Correction: cDNA-AFLP analysis reveals differential gene expression in compatible interaction of wheat challenged with Puccinia striiformis f. sp. tritici

**DOI:** 10.1186/1471-2164-14-671

**Published:** 2013-10-07

**Authors:** Xiaojie Wang, Chunlei Tang, Gang Zhang, Yingchun Li, Chenfang Wang, Bo Liu, Zhipeng Qu, Jie Zhao, Qingmei Han, Lili Huang, Xianming Chen, Zhensheng Kang

**Affiliations:** 1 College of Plant Protection and Shaanxi Key Laboratory of Molecular Biology for Agriculture, Northwest A&F University, Yangling, Shaanxi 712100, PR China; 2 USDA-ARS and Department of Plant Pathology, Washington State University, Pullman, WA 99164-6430, USA

## Text

After the publication of this work [1], we became aware of the fact that the GenBank accessions listed in column 2 of Table 1 and Additional File 1 are incorrect. The EF# accession numbers should be prefixed with FF# and not with EF#. The corrected Table 1 is provided below. The corrected additional file can be accessed via the additional file contained in this article.

**Table 1:** **Transcript derived fragments (TDFs) from wheat leaves infected by *Puccinia striiformis *f. sp. *tritici *with homologies to genes in *P. graminis *f. sp. *tritici***

**TDF**	**Accession No.**	**Size (bp)**	***P graminis *f. sp. *tritici *clones**	**E-value**
PST_72-1-2b	FF339818	235	PGTG_02587|Glycine dehydrogenase	7e-15
PST_C40	FF339683	311	PGTG_04973 | Fructose-1,6-bisphosphatase	1e-28
PST_C37^a^	FF339680	294	PGTG_01121| UDP-glucuronic acid decarboxylase	3e-22
PST_C38	FF339681	265	PGTG_15605| ATP synthase subunit alpha	3e-15
PST_C87	FF339730	237	PGTG_04870| ATP synthase subunit beta	1e-15
PST_C59^a^	FF339702	643	PGTG_06894 |NADH-quinone oxidoreductase chain 3	3e-74
PST_315-3^a^	FF339797	407	PGTG_16250| possible glycosyl transferase	1e-16
PST_84-3b^a^	FF339823	410	PGTG_13068|Conserved hypothetical protein	7e-25
PST_C81	FF339724	318	PGTG_08200 |Vesicular-fusion protein SEC17	3e-14
PST_C16	FF339660	366	PGTG_14848|Conserved hypothetical protein	2e-09
PST_C88	FF339731	216	PGTG_14274|Plasma membrane proteolipid 3	2e-12
PST_68b-1	FF339813	259	PGTG_18059|NADH-quinone oxidoreductase	4e-06
PST_68b-3^a^	FF339814	257	PGTG_18059|NADH-quinone oxidoreductase	7e-16
PST_C101^a^	FF339744	586	PGTG_07295|Conserved hypothetical protein	1e-21
PST_315-4^a^	FF339798	404	PGTG_10913|Predicted protein	1e-21
PST_C86^a^	FF339729	436	PGTG_15782|Hypothetical protein	8e-32
PST_C83^a^	FF339726	414	PGTG_02587 | Glycine dehydrogenase	7e-33
PST_C73	FF339716	249	PGTG_13068 | Conserved hypothetical protein	2e-24

^a^ These genes are confirmed to be of stripe rust fungus origin by sequencing.

Wang *et al. BMC Genomics* 2009 **10**:289 http://www.biomedcentral.com/1471-2164/10/289.

## Supplementary Material

Additional file 1 **Transcript derived fragments (TDFs) from *Puccinia striiformis* f. sp. *tritici* infected wheat leaves with altered expression patterns and their closest matches in the GenBank database.** Click here for file
