# Supplementary material for: Correction: cDNA-AFLP analysis reveals differential gene expression in compatible interaction of wheat challenged with Puccinia striiformis f. sp. tritici
Source: BMC Genomics. 2013 Oct 7;14:671. doi: 10.1186/1471-2164-14-671 (PMC3850898; doi:10.1186/1471-2164-14-671)
Supplement: Additional file 1 — Transcript derived fragments (TDFs) from Puccinia striiformis f. sp. tritici infected wheat leaves with altered expression patterns and their closest matches in the GenBank database. [file 1471-2164-14-671-S1.doc]

**Additional file 1: Transcript derived fragments (TDFs) from *Puccinia striiformis* f. sp. *tritici* infected wheat leaves with altered expression patterns and their closest matches in the GenBank database**

| TDF | Accession No. | Size  (bp) | Closest to database match | E-value | Expression |
| --- | --- | --- | --- | --- | --- |
| 1. Metabolism | | | | | |
| PST_C70 | FF339713 | 369 | gb|AAL58883.1|methylthioadenosine/S-adenosylhomocysteine nucleosidase [*Oryza sativa*] | 2.00E-19 | Up |
| PST_72-1-2b | FF339818 | 235 | gb|AAB82711.1| glycine decarboxylase P subunit[*Tritordeum* ] | 2.00E-32 | Up |
| PST_312-5 | FF339796 | 337 | gb|AAL58883.1|methylthioadenosine/S-adenosylhomocysteine nucleosidase [*Oryza sativa*] | 2.00E-17 | Up |
| PST_315-3 | FF339797 | 407 | ref|NP_949287.1| possible glycosyl transferase [*Rhodopseudomonas palustris* ] | 1.00E-16 | Up |
| PST_52-5c | FF339809 | 195 | emb|CAA63139.1|aminolevulinate dehydratase [*Hordeum vulgare*] | 2.00E-12 | Down |
| PST_304-4 | FF339747 | 321 | ref|XP_464920.1|Peroxisomal fatty acid beta-oxidation  multifunction nal protein [*Oryza sativa* ] | 8.00E-38 | Down |
| PST_C83 | FF339726 | 414 | gb|AAB82711.1| glycine decarboxylase P subunit [*Tritordeum*] | 2.00E-59 | Up |
| PST_C86 | FF339729 | 436 | ref|XP_749106.1| lipase [*Aspergillus fumigatus*] | 5.00E-13 | Up |
| PST_C40 | FF339683 | 311 | emb|CAB46084.1| fructose-1,6-bisphosphatase [*Pisum sativum*] | 3.00E-44 | Up |
| 2. Energy | | | | | |
| PST_C4 | FF339648 | 291 | gb|AAV33287.1|ribulose-1,5-bisphosphatecarboxylase/oxygenase large subunit [*Brachypodium pinnatum*] | 1.00E-45 | Up |
| PST_C28 | FF339671 | 523 | gb|AAU04826.1|ribulose-1,5-bisphosphatecarboxylase/oxygenase large subunit [*Bromus arenarius*] | 1.00E-86 | Up |
| PST_C18 | FF339661 | 667 | emb|CAA59281.1| firefly luciferase [*Photinus pyralis*] | 1.00E-91 | Down |
| PST_C37 | FF339680 | 294 | gb|AAT80326.1|UDP-D-glucuronatedecarboxylase [*Hordeum*] | 3.00E-39 | Down |
| PST_C38 | FF339681 | 265 | ref|NP_114256.1| ATP synthase CF1 alpha chain [*Triticum aestivum*] | 2.00E-35 | Down |
| PST_C43 | FF339686 | 415 | gb|AAM88439.1| putative Rieske Fe-S precursor protein [*Triticum aestivum*] | 1.00E-65 | Down |
| PST_C46 | FF339689 | 493 | gb|AAV39613.1| carotenoid cleavage dioxygenase [*Zea mays*] | 4.00E-56 | Up |
| PST_C48 | FF339691 | 463 | gb|AAC15992.1| chlorophyll a/b binding protein [*Oryza sativa*] | 2.00E-73 | Down |
| PST_C53 | FF339696 | 192 | emb|CAA44888.1| chlorophyll a/b binding protein precursor[*Zea mays*] | 5.00E-08 | Down |
| PST_C60 | FF339703 | 385 | ref|NP_917313.1| NADP-specific isocitrate dehydrogenase [*Oryza sativa* ] | 2.00E-47 | Up |
| PST_C87 | FF339730 | 237 | emb|CAA25114.1| ATPase, beta subunit [*Hordeum vulgare*] | 6.00E-28 | Down |
| PST_305-4 | FF339749 | 307 | sp|P26667| Ribulose bisphosphate carboxylase small chain[*Triticum aestivum*] | 7.00E-07 | Down |
| PST_C50 | FF339693 | 417 | sp|P40880| Carbonic anhydrase, chloroplast precursor (*Carbonate dehydratase*) carbonic anhydrase | 1.00E-46 | Down |
| PST_C59 | FF339702 | 643 | ref|YP_497565.1|NADH-quinone oxidoreductase, chain G [*Novosphingobium aromaticivorans* ] | e-109 | Up |
| PST_C66 | FF339709 | 338 | emb|CAG25595.1| putative rubisco small subunit [*Triticum turgidum*] | 3.00E-07 | Down |
| PST_C96 | FF339739 | 421 | emb|CAA25058.1|ribulosebisphosphate carboxylase [*Triticum aestivum*] | 2.00E-07 | Down |
| PST_63c-3 | FF339812 | 348 | emb|CAA25058.1|ribulosebisphosphate carboxylase [*Triticum aestivum*] | 7.00E-07 | Down |
| PST_289-5 | FF339789 | 453 | sp|P23993|PSAL_HORVU Photosystem I reaction centersubunit XI | 7.00E-47 | Down |
| PST_17-5 | FF339761 | 333 | gb|AAG04943.1|probable cytochrome oxidase subunit(cbb3-type) [*Pseudomonas aeruginosa*] | 2.00E-55 | Up |
| PST_328-5 | FF339801 | 338 | gb|AAG04943.1|probable cytochrome oxidase subunit(cbb3-type) [*Pseudomonas aeruginosa*] | 2.00E-73 | Up |
| PST_C24 | FF339667 | 153 | ref|XP_467296.1|phosphoribulokinase precursor [*Oryza sativa*] | 5.00E-16 | Down |
| PST_C75 | FF339718 | 416 | ref|XP_478627.1|putative Oxygen-evolving enhancer protein [*Oryza sativa*] | 5.00E-22 | Up |
| PST_C84 | FF339727 | 391 | pir||T02955 probable cytochrome P450 monooxygenase [maize] | 9.00E-26 | Up |
| ST_272-2 | FF339776 | 335 | pir||T02955 probable cytochrome P450 monooxygenase[maize] | 9.00E-63 | Up |
| 3. Cell growth/division | | | | | |
| PST_C71 | FF339714 | 359 | gb|AAS48884.1| expansin EXPB7 [*Triticum aestivum*] | 4.00E-42 | Up |
| 4. Transcription | | | | | |
| PST_C58 | FF339701 | 425 | gb|ABA97622.2| RNA binding protein, putative,expressed [*Oryza sativa*] | 9.00E-63 | Up |
| PST_C68 | FF339711 | 231 | gb|AAK13589.1| rRNA intron-encoded homing endonuclease [*Oryza sativa*] | 4.00E-19 | Up |
| PST_C92 | FF339735 | 483 | gb|AAK13589.1| rRNA intron-encoded homing endonuclease [*Oryza sativa*] | 5.00E-15 | Up |
| PST_289-2 | FF339787 | 462 | gb|AAF05865.1| NAM-like protein [*Arabidopsis thaliana*] | 1.00E-13 | Up |
| 5. Protein synthesis | | | | | |
| PST_260-1 | FF339764 | 215 | gb|AAW50981.1| ribosomal protein L6 [*Triticum aestivum*] | 6.00E-24 | Down |
| PST_C39 | FF339682 | 271 | gb|AAV31343.1| putative chloroplast ribosomal proteinL1 [*Oryza sativa*] | 9.00E-63 | Down |
| PST_C34 | FF339677 | 230 | gb|ABA97622.2| RNA binding protein, putative, expressed [*Oryza sativa*] | 5.00E-19 | Up |
| 6. Protein destination and storage | | | | | |
| PST_C73 | FF339716 | 249 | gb|AAL77200.1| ubiquitin [*Oryza sativa*] | 7.00E-23 | Down |
| PST_84-3b | FF339823 | 410 | gb|AAL77200.1| ubiquitin [*Oryza sativa*] | 2.00E-23 | Down |
| 7. Transporters | | | | | |
| PST_C5 | FF339649 | 338 | emb|CAD59448.1| MRP-like ABC transporter [*Oryza sativa*] | 8.00E-06 | Up |
| PST_C33 | FF339676 | 208 | emb|CAD59448.1| MRP-like ABC transporter [*Oryza sativa*] | 8.00E-06 | Up |
| PST_289-3 | FF339788 | 480 | gi|50939843| putative potassium transporter [*Oryzas ativa*] | 5.00E-59 | Up |
| 8. Intracellular traffic | | | | | |
| PST_298-2 | FF339791 | 202 | ref|NP_920969.1| putative atpE; ATPase epsilon subunitfrom chromosome 10 chloroplast insertion [*Oryza sativa*] | 3.00E-15 | Up |
| PST_C81 | FF339724 | 318 | ref|XP_481268.1| putative alpha-soluble NSF attachmentprotein [*Oryza sativa* ] | 7.00E-23 | Up |
| PST_73b-5 | FF339821 | 130 | ref|NP_910644.1| putative vacuolar ATP synthase subunitH [*Oryza sativa*] | 2.00E-11 | Up |
| PST_275-4 | FF339779 | 413 | ref|XP_478377.1|putative chloroplast (H(+)-transporting two-sector ATPase/F(1)-ATPase/ATPC1) [*Oryza sativa*] | 4.00E-44 | Up |
| 9. Cell structure | | | | | |
| PST_335-1 | FF339802 | 406 | ref|ZP_00817021.1| flagellin type B [*Marinobacter aquaeolei*] | 2.00E-16 | Down |
| PST_C29 | FF339672 | 324 | emb|CAJ83813.1| CHK1 checkpoint homolog [*Xenopus tropicalis*] | 2.00E-08 | Up |
| PST_C30 | FF339673 | 238 | emb|CAJ83813.1| CHK1 checkpoint homolog[*Xenopus tropicalis*] | 2.00E-08 | Up |
| 10. Signal transduction | | | | | |
| PST_C61 | FF339704 | 388 | emb|CAD42640.1| putative MAPKK kinase [*Hordeum vulgare*] | 4.00E-24 | Up |
| PST_298-3 | FF339792 | 204 | ref|XP_477491.1| putative receptor-type protein kinaseLRK1 [*Oryza sativa*] | 3.00E-17 | Up |
| PST_C16 | FF339660 | 366 | dbj|BAD37980.1| phosphatase-like [*Oryza sativa*] | 1.00E-24 | Up |
| PST_C12 | FF339656 | 676 | gb|ABF95153.1|Variant SH3 domain containing protein, [*Oryza sativa*] | 4.00E-63 | Up |
| PST_70-5b | FF339816 | 242 | gb|AAD10483.1| p34cdc2 [*Triticum aestivum*] | 7.00E-07 | Up |
| PST_261-1 | FF339766 | 196 | emb|CAC59976.1| pollen signalling protein with adenylylcyclase activity [*Zea mays*] | 5.00E-19 | Down |
| PST_143-7 | FF339828 | 571 | AAF75093.1|Contains similarity to a receptor-like serine/threonine kinase [*Arabidopsis thaliana*] | 7.00E-82 | Up |
| PST_274-5 | FF339829 | 716 | XP_550059.1|putative receptor serine/threonine kinase PR5K [*Oryza sativa*] | 1.00E-11 | Up |
| PST_576-6 | FF339830 | 568 | NP_916407.1| putative receptor kinase [*Oryza sativa*] | 7.00E-77 | Up |
| PST_106B-4 | FF339751 | 535 | gi|72006603 similar to very largeG-protein coupled receptor 1 [*Strongylocentrotus purpuratus*] | 1.00E-08 | Up |
| 11. Disease/defence | | | | | |
| PST_C44 | FF339687 | 378 | emb|CAA80493.1| (1,3;1,4) beta glucanase [*Triticum aestivum*] | 6.00E-60 | Up |
| PST_C6 | FF339650 | 343 | gb|AAW52718.1| peroxidase 4 [*Triticum monococcum*] | 2.00E-43 | Up |
| PST_C36 | FF339679 | 262 | ref|XP_550224.1|verticillium wilt disease resistance protein -like [*Oryza sativa*] | 2.00E-16 | Down |
| PST_C65 | FF339708 | 290 | gb|AAR25995.1|putative senescence-associated protein [*Pyrus communis*] | 2.00E-23 | Up |
| PST_267-4 | FF339771 | 263 | ref|XP_550224.1|verticillium wilt disease resistanceprotein-like [*Oryza sativa*] | 7.00E-83 | Down |
| PST_C88 | FF339731 | 216 | dbj|BAE07207.1|salt-stress induced hydrophobic peptide [*Lophopyrum elongatum*] | 7.00E-23 | Up |
| PST_C56 | FF339699 | 376 | gb|AAA58585.2| hemolysin [*Acanthamoeba polyphaga*] | 2.00E-59 | Up |
| PST_68b-1 | FF339813 | 259 | gb|AAW78582.1| quinone reductase 2 [*Triticum monococcum*] | 4.00E-20 | Up |
| PST_68b-3 | FF339814 | 257 | gb|AAW78582.1| quinone reductase 2 [*Triticum monococcum*] | 9.00E-21 | Up |
| PST_266-3 | FF339768 | 262 | gb|AAP04433.1| putative protease inhibitor [*Hordeum vulgare*] | 2.00E-18 | Up |
| PST_266-4 | FF339769 | 262 | gb|AAP04433.1|subtilisin-chymotrypsin inhibitor 2 [*Hordeum vulgare*] | 9.00E-18 | Up |
| 12. No hit | | | | | |
| PST_C3 | FF339647 | 251 | No hits found | - | Down |
| PST_C7 | FF339651 | 320 | No hits found | - | Down |
| PST_C9 | FF339653 | 203 | No hits found | - | Down |
| PST_C13 | FF339657 | 233 | No hits found | - | Up |
| PST_C14 | FF339658 | 317 | No hits found | - | Up |
| PST_C15 | FF339659 | 106 | No hits found | - | Down |
| PST_C20 | FF339663 | 302 | No hits found | - | Down |
| PST_C21 | FF339664 | 308 | No hits found | - | Up |
| PST_C23 | FF339666 | 243 | No hits found | - | Down |
| PST_C25 | FF339668 | 266 | No hits found | - | Down |
| PST_C26 | FF339669 | 266 | No hits found | - | Down |
| PST_C31 | FF339674 | 189 | No hits found | - | Up |
| PST_C32 | FF339675 | 212 | No hits found | - | Up |
| PST_C42 | FF339685 | 355 | No hits found | - | Up |
| PST_C47 | FF339690 | 328 | No hits found | - | Down |
| PST_C49 | FF339692 | 237 | No hits found | - | Down |
| PST_C51 | FF339694 | 203 | No hits found | - | Down |
| PST_C55 | FF339698 | 152 | No hits found | - | Down |
| PST_C63 | FF339706 | 156 | No hits found | - | Down |
| PST_C67 | FF339710 | 264 | No hits found | - | Up |
| PST_C69 | FF339712 | 320 | No hits found | - | Down |
| PST_C72 | FF339715 | 356 | No hits found | - | Down |
| PST_C74 | FF339717 | 250 | No hits found | - | Down |
| PST_C76 | FF339719 | 150 | No hits found | - | Down |
| PST_C79 | FF339722 | 435 | No hits found | - | Down |
| PST_C80 | FF339723 | 268 | No hits found | - | Up |
| PST_C85 | FF339728 | 181 | No hits found | - | Down |
| PST_C89 | FF339732 | 459 | No hits found | - | Down |
| PST_C93 | FF339736 | 267 | No hits found | - | Up |
| PST_C94 | FF339737 | 239 | No hits found | - | Down |
| PST_C101 | FF339744 | 586 | No hits found | - | Down |
| PST_296-1 | FF339746 | 187 | No hits found | - | Down |
| PST_305-5 | FF339750 | 321 | No hits found | - | Down |
| PST_115s-1 | FF339752 | 190 | No hits found | - | Down |
| PST_115s-3 | FF339753 | 185 | No hits found | - | Down |
| PST_118-3 | FF339754 | 141 | No hits found | - | Down |
| PST_154-5 | FF339755 | 137 | No hits found | - | Down |
| ST_162-1-1 | FF339756 | 243 | No hits found | - | Down |
| PST_162-1 | FF339757 | 176 | No hits found | - | Down |
| PST_168x-3 | FF339759 | 214 | No hits found | - | Down |
| PST_168x-5 | FF339760 | 327 | No hits found | - | Down |
| PST_258-3 | FF339763 | 169 | No hits found | - | Up |
| PST_260-2 | FF339765 | 193 | No hits found | - | Up |
| PST_263-5 | FF339767 | 211 | No hits found | - | Down |
| ST_267-1-5 | FF339770 | 263 | No hits found | - | Down |
| PST_269-4 | FF339773 | 290 | No hits found | - | Down |
| PST_270-3 | FF339774 | 310 | No hits found | - | Down |
| PST_273-2 | FF339777 | 357 | No hits found | - | Down |
| PST_282-1 | FF339781 | 164 | No hits found | - | Down |
| PST_282-4 | FF339782 | 164 | No hits found | - | Down |
| PST_285-2 | FF339783 | 364 | No hits found | - | Up |
| PST_285-3 | FF339784 | 354 | No hits found | - | Down |
| PST_288-2 | FF339785 | 377 | No hits found | - | Down |
| PST_297-5 | FF339790 | 193 | No hits found | - | Down |
| PST_309-2 | FF339794 | 319 | No hits found | - | Down |
| PST_315-4 | FF339798 | 404 | No hits found | - | Down |
| PST_325-1 | FF339799 | 123 | No hits found | - | Down |
| PST_325-2 | FF339800 | 130 | No hits found | - | Down |
| PST_336-2 | FF339804 | 271 | No hits found | - | Up |
| ST_342-1-4 | FF339806 | 282 | No hits found | - | Down |
| PST_41-5c | FF339807 | 172 | No hits found | - | Down |
| PST_63b-1 | FF339811 | 180 | No hits found | - | Down |
| PST_7-2 | FF339815 | 182 | No hits found | - | Up |
| PST_71b-5 | FF339817 | 121 | No hits found | - | Down |
| PST_92-4 | FF339824 | 104 | No hits found | - | Down |
| PST_92b-5 | FF339825 | 206 | No hits found | - | Down |
| PST_96-10 | FF339827 | 168 | No hits found | - | Up |
| 13. Unknown protein | | | | | |
| PST_C41 | FF339684 | 566 | gb|AAV25649.1| unknown protFin [*Oryza sativa*] | 8.00E-22 | Down |
| PST_C45 | FF339688 | 391 | dbj|BAD54375.1| unknown protein [*Oryza sativa*] | 2.00E-30 | Down |
| PST_279-5 | FF339780 | 328 | dbj|BAD46049.1| unknown protein [*Oryza sativa*] | 2.00E-07 | Down |
| PST_51-1c | FF339808 | 188 | ref|XP_550425.1| unknown protein [*Oryza sativa*] | 3.00E-15 | Up |
| PST_59-5c | FF339810 | 356 | ref|XP_467147.1| unknown protein [*Oryza sativa*] | 3.00E-19 | Down |
| ST_73-1b-4 | FF339819 | 250 | ref|XP_482538.1| unknown protein [*Oryza sativa*] | 2.00E-19 | Down |
| PST_C90 | FF339733 | 450 | gb|AAV44205.1| unknow protein [*Oryza sativa*] | 2.00E-28 | Down |
| PST_73b-4 | FF339820 | 274 | gb|AAH53854.1|Unknown protein[*Homo sapiens*] | 6.00E-06 | Down |
| PST_21-2 | FF339762 | 298 | gb|AAO09931.1| Unknown [*Vibrio vulnificus*] | 2.00E-11 | Up |
| PST_C95 | FF339738 | 251 | ref|XP_475020.1| OSJNBb0093G06.1 [*Oryza sativa*] | 1.00E-29 | Up |
| PST_C97 | FF339740 | 590 | ref|XP_474204.1| OSJNBa0011F23.20 [*Oryza sativa*] | 7.00E-16 | Down |
| ST_269-1-2 | FF339772 | 289 | emb|CAE05443.2| OSJNBa0073E02.3 [*Oryza sativa*] | 3.00E-30 | Down |
| PST_C19 | FF339662 | 447 | gb|AAO74140.1| ORF64c [*Pinus koraiensis*] | 2.00E-17 | Down |
| PST_335-4 | FF339803 | 343 | gb|AAO74140.1| ORF64c [*Pinus koraiensis*] | 1.00E-10 | Down |
| PST_C64 | FF339707 | 165 | gb|AAO74140.1| ORF64c [*Pinus koraiensis*] | 5.00E-06 | Down |
| PST_C35 | FF339678 | 230 | ref|NP_914288.1| P0458E05.17 [*Oryza sativa*] | 8.00E-22 | Down |
| PST_C78 | FF339721 | 304 | ref|NP_917699.1| P0686E09.22 [*Oryza sativa*] | 1.00E-35 | Down |
| PST_C102 | FF339745 | 204 | ref|XP_453837.1| unnamed protein product [*Kluyveromyces lactis*] | 3.00E-12 | Up |
| PST_289-3 | FF339788 | 480 | gb|AAF36491.1| HAK2 [*Hordeum vulgare*] | 8.00E-68 | Down |
| PST_C1 | FF339645 | 471 | ref|XP_430524.1|hypothetical protein [*Gallus gallus*] | 8.00E-06 | Down |
| PST_C2 | FF339646 | 212 | pir|F81737|hypothetical protein [*Chlamydia muridarum*] | 4.00E-10 | Up |
| PST_C8 | FF339652 | 671 | dbj|BAE73006.1| hypothetical protein [*Macacafas cicularis*] | 1.00E-09 | Down |
| PST_C10 | FF339654 | 532 | ref|XP_001067326.1| hypothetical protein[*Rattus norvegicus*] | 1.00E-13 | Down |
| PST_C11 | FF339655 | 259 | ref|YP_173415.1|hypothetical protein [*Nicotiana tabacum*] | 8.00E-22 | Down |
| PST_C22 | FF339665 | 194 | gb|ABE87179.1|hypothetical protein [*Medicago truncatula*] | 2.00E-14 | Down |
| PST_C27 | FF339670 | 319 | ref|ZP_00345902.1| hypothetical protein [*Nostoc punctiforme*] | 2.00E-28 | Up |
| PST_C52 | FF339695 | 514 | gb|ABE87179.1|hypothetical protein [*Medicago truncatula*] | 6.00E-09 | Down |
| PST_C54 | FF339697 | 188 | ref|ZP_00345902.1| hypothetical protein [*Nostoc punctiforme*] | 2.00E-15 | Up |
| PST_C57 | FF339700 | 243 | pir|F81737|hypothetical protein [*Chlamydia muridarum*] | 4.00E-10 | Up |
| PST_C62 | FF339705 | 369 | ref|ZP_00345902.1|hypothetical protein [*Nostoc punctiforme*] | 4.00E-22 | Up |
| PST_C77 | FF339720 | 592 | ref|XP_001067326.1|hypothetical protein[*Rattus norvegicus*] | 3.00E-06 | Down |
| PST_C82 | FF339725 | 778 | ref|ZP_00232118.1|hypothetical protein[*Listeria monocytogenes* str] | 2.00E-28 | Up |
| PST_C98 | FF339741 | 687 | ref|XP_430524.1|hypothetical protein[*Gallus gallus*] | 4.00E-09 | Down |
| PST_C99 | FF339742 | 393 | ref|XP_001067326.1|hypothetical protein[*Rattus norvegicus*] | 8.00E-06 | Down |
| PST_C100 | FF339743 | 441 | ref|XP_430524.1| hypothetical protein [*Gallus gallus*] | 2.00E-06 | Up |
| PST_154-5 | FF339755 | 175 | gb|AAU03684.1| conserved hypothetical protein[*Rickettsia typhi*] | 2.00E-62 | Up |
| ST_168s-3 | FF339758 | 543 | ref|XP_430524.1| hypothetical protein[*Gallus gallus*] | 1.00E-06 | Down |
| PST_274-3 | FF339778 | 221 | pir||F81737 hypothetical protein [*Chlamydia muridarum*] | 6.00E-09 | Up |
| PST_312-2 | FF339795 | 204 | ref|ZP_00569683.1| conserved hypothetical protein[*Frankia*] | 6.00E-09 | Up |
| PST_339-4 | FF339805 | 349 | ref|YP_173415.1| hypothetical protein [*Nicotiana tabacum*] | 2.00E-73 | Down |
| PST_93bx-1 | FF339826 | 520 | ref|XP_430524.1|hypothetical protein [*Gallus gallus*] | 5.00E-06 | Down |
| PST_C91 | FF339734 | 487 | gb|ABA91056.1|expressed protein [*Oryza sativa*] | 7.00E-41 | Down |
| PST_272-1 | FF339775 | 347 | |gb|ABA96147.1| expressed protein [*Oryza sativa*] | 3.00E-12 | Down |
| PST_288-3 | FF339786 | 256 | gb|ABF95764.1| expressed protein [*Oryza sativa*] | 2.00E-09 | Down |
| PST_298-5 | FF339793 | 210 | ref|XP_463045.1| expressed protein [*Oryza sativa*] | 3.00E-12 | Down |
